# Impact of Pandemic Response on Training Experience of Anesthesiology Residents in an Academic Medical Center: A Retrospective Cohort Study

**DOI:** 10.7759/cureus.33500

**Published:** 2023-01-08

**Authors:** Thomas Grissom, Ron E Samet, Caleb B Hodge, Megan G Anders, Bianca M Conti, Jason C Brookman, Douglas G Martz, Caron M Hong, Miranda Gibbons, Peter Rock

**Affiliations:** 1 Department of Anesthesiology, University of Maryland School of Medicine, Baltimore, USA; 2 Department of Anesthesiology, Georgetown University School of Medicine, Washington, USA

**Keywords:** covid 19, accreditation council for graduate medical education (acgme), procedure training, academic anesthesiology, anesthesia residency

## Abstract

Background

The impact of the coronavirus disease 2019 (COVID-19) pandemic substantially altered operations at hospitals that support graduate medical education. We examined the impact of the pandemic on an anesthesiology training program with respect to overall case volume, subspecialty exposure, procedural skill experience, and approaches to airway management.

Methods

Data for this single center, retrospective cohort study came from an Institutional Review Board approved repository for clinical data. Date ranges were divided into the following phases in 2020: Pre-Pandemic (PP), Early Pandemic (EP), Recovery 1 (R1), and Recovery 2 (R2). All periods were compared to the same period from 2019 for case volume, anesthesia provider type, trainee exposure to Accreditation Council for Graduate Medical Education (ACGME) index case categories, airway technique, and patient variables.

Results

15,087 cases were identified, with 5,598 (37.6%) in the PP phase, 1,570 (10.5%) in the EP phase, 1,451 (9.7%) in the R1 phase, and 6,269 (42.1%) in the R2 phase. There was a significant reduction in case volume during the EP phase compared to the corresponding period in 2019 (-55.3%; *P* < .001) that improved but did not return to baseline by the R2 phase (-17.6%; *P* < .001). ACGME required minimum cases were reduced during the EP phase compared to 2019 data for pediatric cases (age < 12 y, -72.1%; *P* < .001 and age < 3 y, -53.5%; *P* < .006) and cardiopulmonary bypass cases (52.3%, *P* < .003). Surgical subspecialty case volumes were significantly reduced in the EP phase except for transplant surgery. By the R2 phase, all subspecialty volumes had recovered except for plastic surgery (14.9 vs. 10.5 cases/week; *P* < .006) and surgical endoscopy (59.2 vs. 40 cases/week; *P* < .001). Use of video laryngoscopy (VL) and rapid sequence induction and intubation (RSII) also increased from the PP to the EP phase (24.6 vs. 79.6%; *P* < .001 and 10.3 vs. 52.3%; *P* < .001, respectively) and remained elevated into the R2 phase (35.2%; *P* < 0.001 and 23.1%; *P* < .001, respectively).

Conclusions

The COVID-19 pandemic produced significant changes in surgical case exposure for a relatively short period. The impact was short-lived, with sufficient remaining time to meet the annual ACGME program minimum case requirements and procedural experiences. The longer-term impact may be a shift towards the increased use of VL and RSII, which became more prevalent during the early phase of the pandemic.

## Introduction

Coronavirus disease 2019 (COVID-19) caused by the severe acute respiratory syndrome coronavirus 2 (SARS-CoV-2) has unquestionably impacted nearly every facet of healthcare delivery on a global scale. Trainees in anesthesiology and other specialties worldwide have been directly affected by common pandemic response measures and their sequelae. The potential consequences of the altered anesthesiology trainee experience have been reviewed [1-7]. One common, early, worldwide response measure was the cessation of non-emergent surgical cases to preserve resources, including personal protective equipment (PPE) and acute care hospital beds. In surgical and anesthesia training, which are experiential by nature and rely heavily on case exposure, fewer surgical cases provide fewer learning opportunities. Multiple investigators have reported the impact of decreased case volume on general surgical and emergency medicine training as well as that in surgical sub-specialties [8-18]. In the United States (US), the Accreditation Council for Graduate Medical Education (ACGME) specifies the expected minimum number for defined category or “index” cases meeting patient or procedure criteria that are required for each trainee to complete during their residency training. It is expected that each trainee will not only meet but surpass these requirements in most instances. The potential exists that altered case volumes during the pandemic may affect achieving those targets, although the impact of this interim case differential on trainee experience may range from being profound to negligible, depending on the institutions’ ability to adjust and rebound to required case volumes.

In addition to reductions in case volume and subspecialty experience, early pandemic response measures also affected trainees through conversion to virtual educational activities, postponed or cancelled examinations, and decreased opportunities supervised procedures [5,19,20]. As with case numbers, the ACGME requires anesthesiology trainees to complete a certain number of supervised procedures, such as spinals, epidurals, and peripheral nerve blocks. Furthermore, airway management recommendations early in the pandemic may have changed the trainee experience by emphasizing specific techniques or attempting to limit trainee exposure to SARS-CoV-2 [21-25]. To gain a more complete understanding of the quantitative early effect of the COVID-19 pandemic on the anesthesia trainee experience, we assessed the impact on case volume, specific index cases and procedures, and general airway management techniques compared to the same period in the previous year in a large US academic anesthesiology department.

## Materials and methods

This is a retrospective cohort study examining the impact of the COVID-19 pandemic on an academic anesthesiology training program. Surgical and anesthesia case data were retrospectively reviewed for the period 1 January to 30 August 2019 and the corresponding period in 2020 for the University of Maryland Medical Center (UMMC) and R Adams Cowley Shock Trauma Center (STC). These facilities are co-located and share many similar resources but have different primary missions. UMMC provides a broad range of surgical specialty support while functioning as the primary adult trauma clinical resource center for Maryland. All anesthesiology trainees rotate through both facilities, with UMMC providing most of the case volume per resident during training. UMMC is a tertiary academic center with an annual case volume of greater than 24,000 in 27 operating rooms (ORs), an obstetrical unit, an endoscopy suite, and multiple non-OR anesthetizing (NORA) locations. STC is an attached facility specializing in trauma, with an annual case volume of greater than 5,500 in nine ORs. There was a total of 52 anesthesiology residents in the anesthesiology training program in 2019 and 46 in 2020. Data for the study was obtained from the Anesthesiology Perioperative Data Warehouse, an institutional review board (IRB) approved repository for clinical data. Institutional IRB approval was obtained with a waiver for written consent.

**Figure 1:**
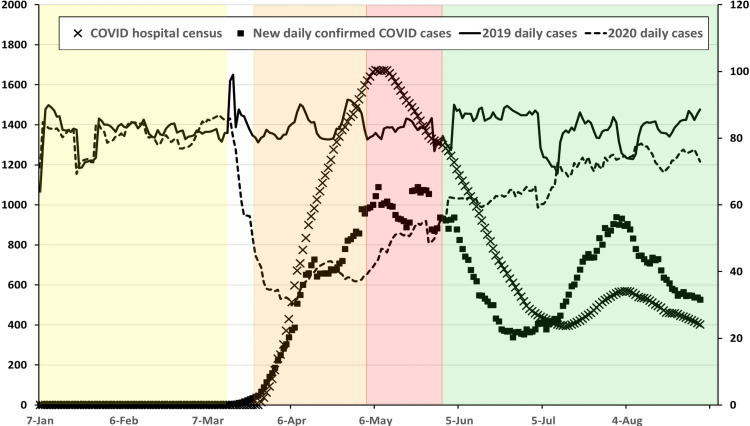
Daily combined operative cases at UMMC and STC for 2019 (solid line) and 2020 (dashed line) from 7 January to 31 August for each year (right axis) shown with the 2020 daily Maryland in-hospital census for patients diagnosed with COVID-19 (X) and daily newly confirmed cases of COVID-19 (solid box) for the same period (left axis). Daily combined operative cases at UMMC and STC for 2019 (solid line) and 2020 (dashed line) from 7 January to 31 August for each year (right axis) shown with the 2020 daily Maryland in-hospital census for patients diagnosed with COVID-19 (X) and daily newly confirmed cases of COVID-19 (solid box) for the same period (left axis). All points are shown as a seven-day moving average. Pandemic phases for 2020 are shown as shaded areas (PP: yellow; EP: orange; R1: red; R2: green). State of Maryland hospital census and daily confirmed cases adapted from https://data.imap.maryland.gov/datasets/mdcovid19-totalcurrentlyhospitalizedacuteandicu/explore and https://data.imap.maryland.gov/datasets/mdcovid19-totalcasesstatewide/explore. COVID-19, coronavirus disease 2019; EP: early pandemic phase; PP: pre-pandemic phase; R1: recovery 1 phase; R2: recovery 2 phase; STC: Shock Trauma Center; UMMC: University of Maryland Medical Center.

On 30 January 2020, the World Health Organization declared the outbreak of SARS-CoV-2 to be a Public Health Emergency of International Concern and COVID-19 a global pandemic on 11 March [26]. The state of Maryland confirmed its first cases of COVID-19 on 5 March, and a local state of emergency was declared at that time (Figure 1). Based on published recommendations and estimates of spread, hospitals reduced or stopped performing elective surgical cases as part of this effort. On 18 March, UMMC and STC decreased the number of utilized general and trauma ORs with the postponement of all elective surgery to improve resource availability for the initial surge in COVID-19 cases. Personnel also began wearing PPE, including an N-95 respirator with a face shield or a powered air-purifying respirator (PAPR), for all aerosolizing procedures regardless of a patient’s COVID-19 status. On 4 May, UMMC and STC began to expand OR availability, including staffing NORA locations for cases deemed “urgent,” with a return to full pre-COVID-19 staffing of all NORA locations with no limitations on case scheduling on 1 June.

To facilitate analysis, the date ranges were divided into the following phases: Pre-Pandemic (PP; 1 January-13 March 2020), Early Pandemic (EP; 23 March-3 May 2020), Recovery 1 (R1; 4 May-30 May 2020), and Recovery 2 (R2; 1 June-31 August 2020). The end date for the PP phase was selected at the time, there were 10 confirmed cases of COVID-19 reported in the state of Maryland, and the first COVID positive patient was admitted to UMMC. The EP phase is defined as the time when all elective cases were canceled at UMMC and STC, and additional restrictions on the number of anesthetizing locations were in place due to newly imposed PPE practices for aerosol-generating procedures (AGP) and limited PPE availability. The period between the PP and EP phases (14 March 2020 to 22 March 2020) was excluded from analysis due to daily changes in practice related to the pandemic; the clinical experience in this period was very heterogeneous. The R1 phase corresponds to liberalized surgical case scheduling (additional anesthetizing locations but less than full schedule), while the R2 phase corresponds to the resumption of normal scheduling and staffing at all anesthetizing locations with appropriate testing and safety precautions in place. Case data collected and analyzed are provided in Table 1.

**Table 1:** Case data collected and analyzed ASA PS: American Society of Anesthesiologists physical status; PUI: Patient under investigation; CRNA: Certified registered nurse anesthetist; STC: Shock Trauma Center; OTO/OMFS: Otorhinolaryngology oral maxillofacial surgery; IR: interventional radiology; OR: Operating room; DL: Direct laryngoscopy; VL: Video laryngoscopy; RSII: Rapid sequence induction and intubation; FOI: Fiberoptic intubation; LMA: Laryngeal mask airway; SRNA: Student registered nurse anesthetist; ACGME: Accreditation College Graduate Medical Education; MAC: Monitored anesthesia care; UMMC: University of Maryland Medical Center.

Case data	Specific parameters						
Patient demographics	Age		Gender		ASA PS class		
COVID-19 status at time of surgery	Negative		Confirmed		PUI		
Case Status	Non-emergent		Emergent (based on addition of “E” for ASA PS)				
Anesthesiology providers assigned to case	Resident		CRNA		Attending		
Surgical subspecialty case	Cardiac	Thoracic	Vascular	Trauma (STC)	OTO/OMFS	Transplant	
	Plastic surgery	Neurosurgery	Orthopedics	Obstetrics	Endoscopy	IR	
Time in OR after surgery end time							
Primary airway management technique	DL	VL	RSII	Mask ventilation at any time	FOI	LMA	
Classification of airway operator	Trainee (fellow, resident, SRNA)		CRNA		Attending		
Extubation attempted in OR	Yes			No			
ACGME index case designation	Pediatric age < 12 year	Pediatric age < 3 year	Regional anesthesia procedure	MAC	Vaginal delivery	Cesarean section	
	Lung isolation	Cardiopulmonary bypass		Major vascular	Pain management procedure		
Case location	UMMC Main OR	Outpatient OR	Obstetric unit	Endoscopy suite	Out-of-OR	STC	

Additionally, reports generated by the ACGME for each academic year (1 July through 30 June) were reviewed for the period encompassing the EP, R1, and R2 phases to assess the pandemic’s impact on the attainment of ACGME minimum defined category cases for trainees enrolled in the program during the respective year.

Statistical analysis

Ages were reported categorically (<3 m, 3 m-3 y, 3-12 y, 12-65 y, >65 y) and continuously. ASA PS was categorized into two groups: ASA 1-2 and ASA 3-5. The proportion of total cases per phase that were COVID-confirmed or PUI was reported as well as the total number of COVID/PUI cases with a resident, attending anesthesiologist, or CRNA assigned. Significance tests were applied to compare PP v. EP phases and PP v. R2 phases using Welch’s t-test for continuous data and χ2 tests for categorical data.

Percent changes in case volume from 2019 to 2020 were reported for surgical locations and ACGME index cases. Classification of major vascular procedures used keyword identification in procedure names (“EVAR”, “TEVAR”, “endovascular aortic”, “aortic aneurysm”, “carotid endarterectomy”, “femoral bypass”, “bypass carotid”,” bypass graft carotid”, and “carotid artery stent”) and excluded cases using cardiopulmonary bypass. Significance tests were applied to compare case volume in each phase with its corresponding period from 2019 using Welch’s t-test.

Percent changes from PP to later phases were reported for surgical subspecialties and airway procedures. Totals for airway procedures represent a percentage of intubations, except for endotracheal tube (ETT) and laryngeal mask airway (LMA), which are represented as a percent of all cases. Patients with extubation taking place in the OR are represented as a percent of cases with documented prior ETT in place or intubated in the OR. Significance tests were applied to compare the average subspecialty case volume per week in PP v. EP phases and PP v. R2 phases using Welch’s t-test. Chi-square tests were used to compare airway management across the different phases.

An α of 0.05 (two-tailed) was used for all performed tests. Significance tests were not applied to COVID-specific demographics. All analyses were conducted in R version 4.0.2 [27].

## Results

**Figure 2:**
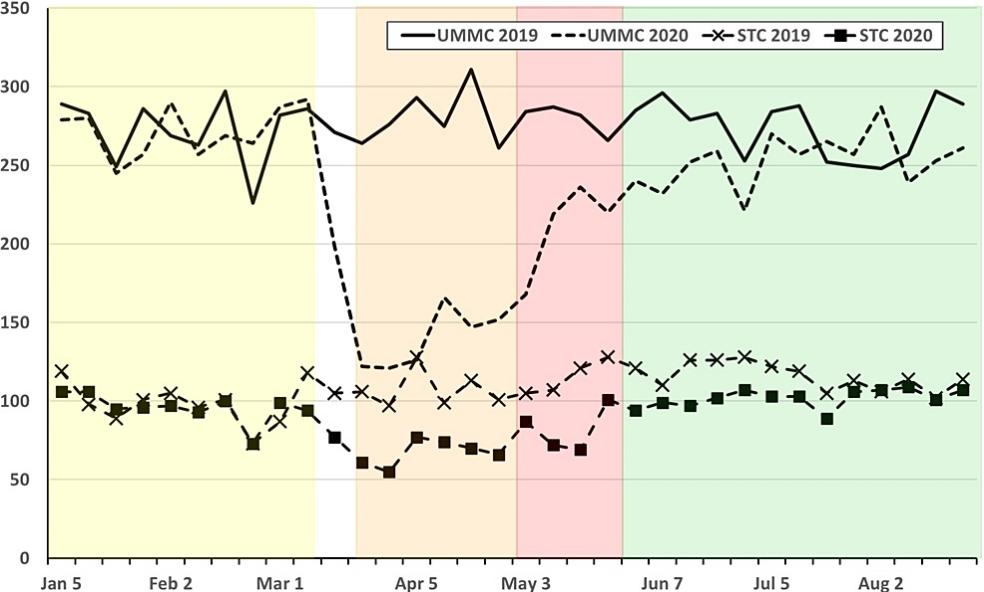
Weekly operative cases at UMMC for 2019 (solid line) and 2020 (dashed line) and STC for 2019 (X) and 2020 (solid box) from 1 January to 31 August for each year. Weekly operative cases at UMMC for 2019 (solid line) and 2020 (dashed line) and STC for 2019 (Ï) and 2020 (n) from 1 January to 31 August for each year. Pandemic phases for 2020 are shown as shaded areas (PP: Yellow; EP: Orange; R1: Red; R2: Green). EP: Early Pandemic Phase; PP: Pre-Pandemic Phase; R1: Recovery 1 Phase; R2: Recovery 2 Phase; STC: Shock Trauma Center; UMMC: University of Maryland Medical Center.

A total of 15,087 cases were performed at UMMC and STC between 1 January and 30 August 2020, with 5,598 (37.6%) in the PP phase, 1,570 (10.5%) in the EP phase, 1,451 (9.7%) in the R1 phase, and 6,269 (42.1%) in the R2 phase (Figure 2). The patient demographics are presented in Table 2. The average patient age was significantly lower in the R2 phase (P < 0.001) compared to the PP phase. There was a slight increase in male patients during the EP phase (P = 0.04), which was resolved by the R2. The percentage of patients with an ASA PS 1 or 2 was higher in the EP phase compared to the PP phase (P < 0.001).

**Table 2:** Patient demographics and provider assignment distribution. * Totals may not equal 100% due to overlapping assignments and alternative providers not shown. Values are mean (SD), n (percentage of total cases or cases by site), or average cases per week. ASA PS: American Society of Anesthesiologists Physical Status; COVID-19: Coronavirus Disease 2019; CRNA: Certified Registered Nurse Anesthetist; EP: Early Pandemic Phase; OR: Operating Room; PP: Pre-Pandemic Phase; PUI: Patient Under Investigation; R1: Recovery 1 Phase; R2: Recovery 2 Phase; SD: Standard Deviation; STC, Shock Trauma Center; UMMC, University of Maryland Medical Center.

	Phase				P-value	
	PP (n=5598)	EP (n=1570)	R1 (n=1451)	R2 (n=6269)	PP vs. EP	PP vs. R2
Cases by site						
UMMC-all locations	4632 (82.7%)	1165 (74.2%)	1124 (77.5%)	4946 (78.9%)	--	--
STC	966 (17.3%)	405 (25.8%)	327 (22.5%)	1323 (21.1%)	--	--
Age (y)	47.6 (23.2)	47.9 (21.1)	46.6 (22.3)	45.7 (23.2)	0.591	< .001>
Average cases/week by age						
< 3 m	6.5	5.8	4.8	5.0	0.685	0.247
3 m-3 y	22.4	6.2	11.3	19.7	<0.001	0.134
3-12 y	27.8	4.7	12.8	26.7	<0.001	0.621
12-65 y	351.8	183.3	248.0	313.6	<0.001	<0.001
> 65 y	151.3	61.7	86.00	117.2	<0.001	<0.001
Male	2924 (52.2%)	866 (55.2%)	802 (55.3%)	3323 (53%)	0.040	0.399
ASA PS classification 1 and 2	1744 (31.2%)	356 (22.7%)	394 (27.2%)	1876 (29.9%)	<0.001	0.147
Classified as emergent by site						
UMMC-main ORs only	237 (8.9%)	127 (15.2%)	94 (11.2%)	312 (9.5%)	<0.001	0.415
UMMC-all locations	506 (10.9%)	257 (22.1%)	188 (16.7%)	678 (13.7%)	<0.001	<0.001
STC	182 (18.8%)	104 (25.7%)	81 (24.8%)	296 (22.4%)	0.005	0.040
Assigned to case^*^						
Resident	2212 (39.5%)	636 (40.5%)	643 (44.3%)	2714 (43.3%)	0.476	<0.001
CRNA	3324 (59.4%)	931 (59.3%)	823 (56.7%)	3511 (56%)	0.955	<0.001
Attending	169 (3.0%)	23 (1.5%)	23 (1.6%)	117 (1.9%)	<0.001	<0.001
COVID-19/PUI cases	0 (0)	180 (11.5%)	104 (7.2%)	199 (3.2%)	--	--
COVID-19/PUI cases by provider^*^						
Resident	0	66 (4.2%)	38 (2.6%)	49 (0.8%)	--	--
CRNA	0	109 (7.0%)	68 (4.7%)	148 (2.4%)	--	--
Time in OR after surgery end (min)	14.9 (11.5)	26.1 (18.0)	27.1 (16.8)	17.5 (12.8)	<0.001	<0.001

**Figure 3:**
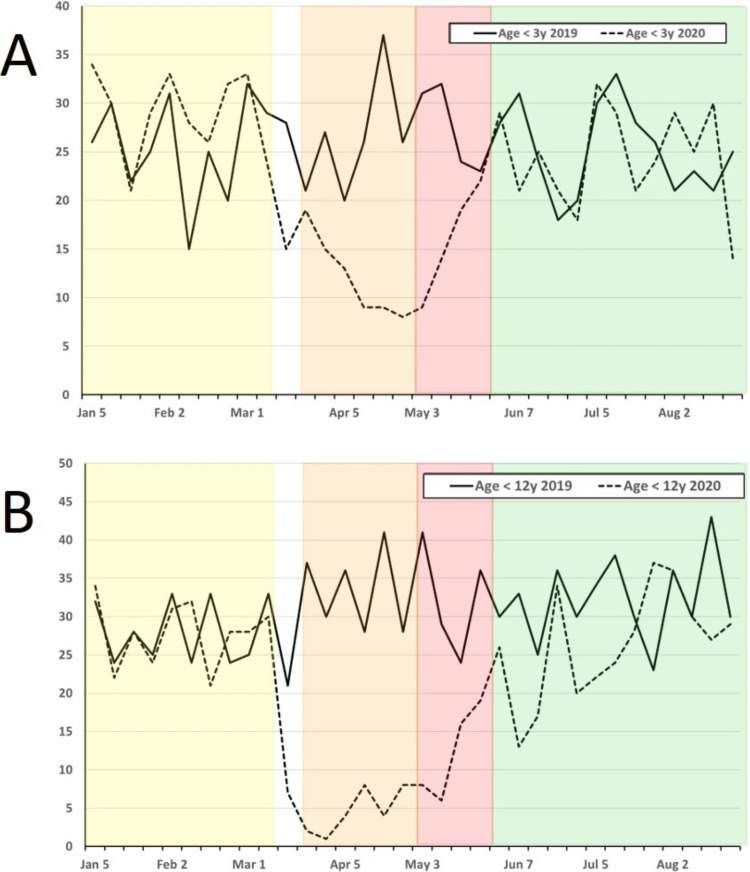
Weekly combined operative cases at UMMC and STC for 2019 (solid line) and 2020 (dashed line) from 1 January to 31 August for each year for patients: A) age < 3 years and B) age < 12 years. Weekly combined operative cases at UMMC and STC for 2019 (solid line) and 2020 (dashed line) from 1 January to 31 August for each year for patients: A) age < 3 years and B) age < 12 years. Age ranges reflect ACGME index case cutoffs for minimum case requirements. Pandemic phases for 2020 are shown as shaded areas (PP, Yellow; EP, Orange; R1, Red; R2, Green). ACGME: Accreditation Council for Graduate Medical Education; EP: Early Pandemic Phase; PP: Pre-Pandemic Phase; R1: Recovery 1 Phase; R2, Recovery 2 Phase; STC: Shock Trauma Center; UMMC: University of Maryland Medical Center.

**Figure 4:**
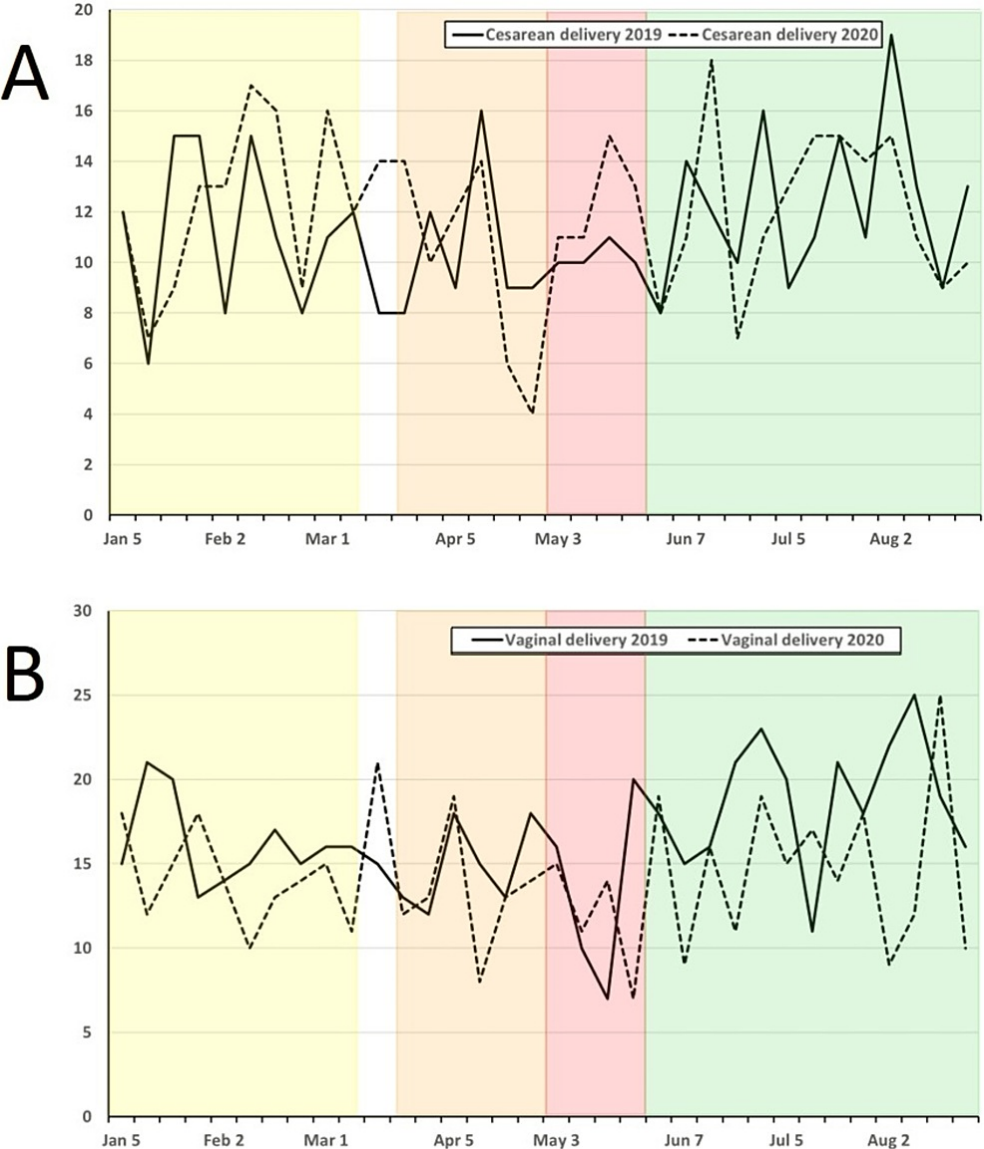
Weekly combined operative cases at UMMC and STC for 2019 (solid line) and 2020 (dashed line) from 1 January to 31 August for each year for obstetric cases incorporating: A) cesarean and B) vaginal delivery. Weekly combined operative cases at UMMC and STC for 2019 (solid line) and 2020 (dashed line) from 1 January to 31 August for each year for obstetric cases incorporating: A) cesarean and B) vaginal delivery. Cases meet the definition for ACGME index case requirements for obstetric case minimums. Pandemic phases for 2020 are shown as shaded areas (PP: Yellow; EP: Orange; R1: Red; R2: Green). ACGME: Accreditation Council for Graduate Medical Education; EP: Early Pandemic Phase; PP: Pre-Pandemic Phase; R1: Recovery 1 Phase; R2: Recovery 2 Phase; STC: Shock Trauma Center; UMMC: University of Maryland Medical Center.

**Figure 5:**
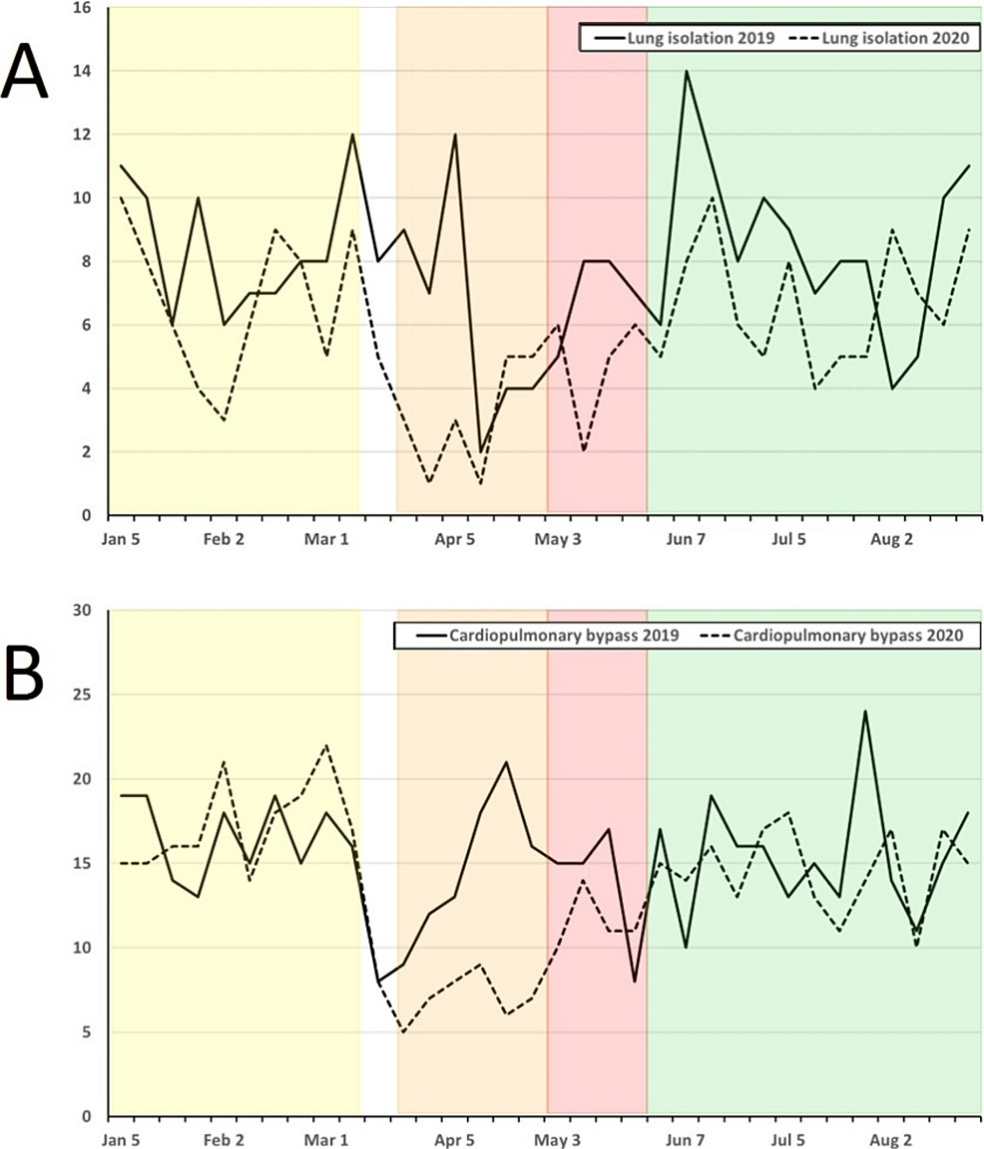
Weekly combined operative cases at UMMC and STC for 2019 (solid line) and 2020 (dashed line) from 1 January to 31 August for each year for cases incorporating: A) lung isolation and B) cardiopulmonary bypass. Weekly combined operative cases at UMMC and STC for 2019 (solid line) and 2020 (dashed line) from 1 January to 31 August for each year for cases incorporating: A) lung isolation and B) cardiopulmonary bypass. Cases meet the definition for ACGME index case requirements for cardiothoracic case minimum. Pandemic phases for 2020 are shown as shaded areas (PP: Yellow; EP: Orange; R1: Red; R2: Green). ACGME: Accreditation Council for Graduate Medical Education; EP: Early Pandemic Phase; PP: Pre-Pandemic Phase; R1: Recovery 1 Phase; R2: Recovery 2 Phase; STC: Shock Trauma Center; UMMC: University of Maryland Medical Center.

**Figure 6:**
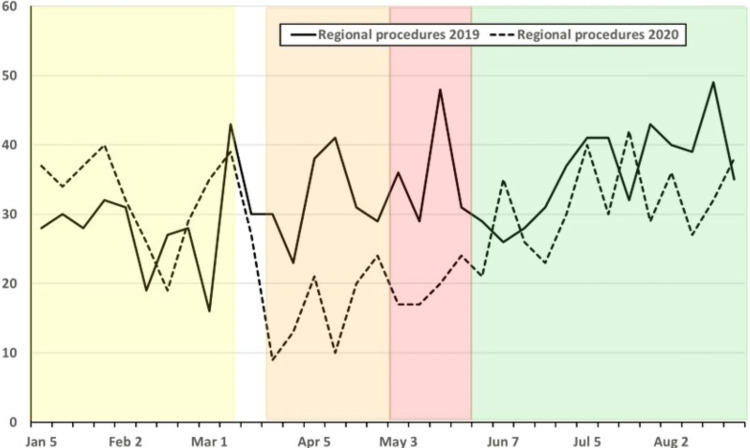
Weekly combined operative cases at UMMC and STC for 2019 (solid line) and 2020 (dashed line) from 1 January to 31 August for each year for the performance of regional anesthesia procedures. Weekly combined operative cases at UMMC and STC for 2019 (solid line) and 2020 (dashed line) from 1 January to 31 August for each year for the performance of regional anesthesia procedures. Cases meet the definition for ACGME index case requirements for case minimums. Pandemic phases for 2020 are shown as shaded areas (PP: Yellow; EP: Orange; R1: Red; R2: Green). ACGME: Accreditation Council for Graduate Medical Education; EP: Early Pandemic Phase; PP: Pre-Pandemic Phase; R1: Recovery 1 Phase; R2: Recovery 2 Phase; STC: Shock Trauma Center; UMMC: University of Maryland Medical Center.

Case volume changed significantly during 2020 compared to 2019, with significant drops in total cases as well as index case volumes for the EP, R1, and R2 phases (Table 3; Figure 2). Comparing the 2020 PP phase to the same period in 2019, there was no significant difference in overall or index case volumes (Table 3; Figure 3). Comparing changes in case volume across the phases (PP vs. EP; PP vs. R2) in 2020, there was no significant change in volume for patients < 3 months old; however, all other age groups showed a significant decline in case volume during the EP phase with this decline persisting into the R2 phase for patients > 12 years old (Table 2; Figure 4). Other index case volumes were variably affected (Table 3; Figures 5, 6). Emergent cases at UMMC increased from 8.9% in the PP phase to 15.2% in the EP phase (P < 0.001). An increase in the percentage of emergent cases was also seen at STC increasing, from 18.8% in the PP phase to 25.7% in the EP phase (P = 0.0054). By the R2 phase, the number of emergent cases at UMMC decreased to 9.5%, which was not significantly different from the PP phase (P = 0.42). STC; however, continued to see a small but significant increase in the percentage of emergent cases (22.4%; P = 0.04).

**Table 3:** Changes in case volume by site and by ACGME index case classification. Values are percentage change from 2019 to 2020 for case volume. ACGME: Accreditation Council for Graduate Medical Education; EP: Early Pandemic Phase; OR: Operating Room; PP: Pre-Pandemic Phase; R1: Recovery 1 Phase; R2: Recovery 2 Phase; STC: Shock Trauma Center; UMMC: University of Maryland Medical Center.

	Change in volume 2019 to 2020 for equivalent period				P value			
	PP	EP	R1	R2	PP	EP	R1	R2
Case volume (%)								
Total	-1.8	-55.3	-37.4	-17.6	0.493	<0.001	<0.001	<0.001
UMMC-main ORs	-2.9	-50.0	-25.0	-7.7	0.426	<0.001	0.012	0.009
UMMC-outpatient ORs	-11.1	-50.0	-10.6	-11.9	0.046	<0.001	0.286	0.123
UMMC-obstetric unit	-5.9	-10.8	-7.5	-12.8	0.367	0.127	0.581	0.067
UMMC-endoscopy	0.1	-97.5	-100.0	-52.8	0.985	<0.001	<0.001	<0.001
UMMC-out-of-OR	24.0	-81.8	-52.1	-34.5	0.016	<0.001	0.023	<0.001
STC OR	-3.7	-37.7	-29.1	-12.1	0.358	<0.001	0.013	<0.001
Index case volume (%)								
Pediatrics (< 12 y)	5.8	-72.1	-51.9	-11.2	0.315	<0.001	0.008	0.055
Pediatrics (< 3 y)	12.9	-53.5	-42.9	-1.8	0.191	0.006	0.013	0.814
Regional procedures	20.5	-42.3	-54.5	-8.7	0.184	0.053	0.017	0.368
Vaginal delivery	-17.5	-9.0	-19.6	-21.2	0.065	0.358	0.455	0.022
Cesarean delivery	12.6	-10.8	27.5	-3.1	0.453	0.558	0.048	0.754
Lung isolation	-19.3	-50.0	-35.7	-21.6	0.107	0.066	0.022	0.044
Cardiopulmonary bypass	-2.9	-52.3	-19.3	-6.4	0.759	0.003	0.174	0.403
Major vascular	8.9	-50.6	27.8	8.6	0.728	0.050	0.495	0.669
Pain procedures	-3.2	-43.2	-4.0	-25.5	0.569	0.022	0.746	<0.001

In the PP phase, participation in the case by a CRNA, resident, or solo attending anesthesiologist was 59.4%, 39.5%, and 3.0%, respectively (Table 2). The total exceeds 100% due to handoffs between different provider types for some portion of the case. Going into the EP phase, the only significant change in the performing provider percentage was seen in the solo anesthesiologist group, which decreased from personally performing 3.0% to 1.5% of cases (P < 0.001). This decrease was maintained through to the R2 phase, where anesthesiologists were only personally performing 1.9% of cases, still representing a significant decrease from the PP phase (P < 0.001). During the R2 phase, there was an increase in resident case participation with a corresponding reduction in CRNA case involvement.

We also assessed the amount of time patients spent in the OR after the completion of surgery as an indicator of the impact of pandemic response measures on metrics such as time to extubation, time spent in the OR after extubation, and time to prepare for patient transport. The average time spent in the OR after surgical completion during the PP phase was 14.9 ± 11.5 min. In the EP phase, this time increased to 26.1 ± 18.0 min (86.7% increase, P < 0.001), and by the R2 phase, it had decreased back down to 17.5 ± 12.8 minutes which was still a statistically significant increase in duration compared to the PP phase (18.9% increase; P = 0.033).

The surgical subspecialty case data is presented in Table 4 using the same periods to report the average number of cases per week. Case volumes in all these subspecialties, except for transplant, showed significant decreases between the PP and EP phases. The most heavily impacted subspecialties during the EP phase were plastic surgery (-77.6%; P < 0.001), endoscopy (-73%, P < 0.001), interventional radiology (-58.7%, P < 0.001), cardiac surgery (-57.9%; P < 0.001), and vascular surgery (56.7%; P < 0.001). The least affected service was obstetrics (-14.7%, P = 0.032). Transplant surgery saw a decrease of 24.8%, but due to the low number of total cases, this was not statistically significant (P = 0.26). In the R2 phase, all service lines experienced an increase from the EP phase as elective cases resumed to a near PP capacity. Only plastic surgery, interventional radiology, and endoscopy remained significantly below PP phase levels through to the R2 phase. A “rebound” phenomenon where case volumes significantly increase upon resumption of elective cases to levels above PP levels was not seen.

**Table 4:** Case volume by specialty across all phases during 2020. * Represents trauma and other cases from STC operating rooms. Values are average cases per week (percentage change from the PP phase). EP: Early Pandemic Phase; OMFS: Oral Maxillofacial Surgery; OTO: Otorhinolaryngology; PP: Pre-Pandemic Phase; PUI: Patient Under Investigation; R1, Recovery 1 Phase; R2, Recovery 2 Phase; STC, Shock Trauma Center.

Surgical Service	Average case volume per week by phase (% change from PP)				P value	
	PP	EP	R1	R2	PP vs EP	PP vs R2
Cardiac surgery	31.7	13.3 (-57.9)	24.5 (-22.7)	30.5 (-3.9)	<0.001	0.645
Thoracic	5.3	3 (-43.4)	2.5 (-52.8)	5.1 (-4.2)	0.027	0.756
Vascular	25.4	11 (-56.7)	18.8 (-26.2)	25.8 (1.5)	<0.001	0.891
STC*	96.6	67.5 (-30.1)	81.8 (-15.4)	101.8 (5.4)	<0.001	0.116
OTO/OMFS	29.7	18.2 (-38.8)	18.5 (-37.7)	26.4 (-11.2)	<0.001	0.065
Transplant	8.2	6.2 (-24.8)	9.3 (12.8)	6.9 (-15.6)	0.257	0.417
Plastic surgery	14.9	3.3 (-77.6)	7 (-53.0)	10.5 (-29.8)	<0.001	0.006
Neurosurgery	38	23.7 (-37.7)	35 (-7.9)	34 (-10.5)	<0.001	0.121
Orthopedics	52.7	34.3 (-34.9)	43.5 (17.5)	58.4 (10.8)	<0.001	0.1195
Obstetrics	29.5	25.2 (-14.7)	26 (-11.9)	30.7 (4.0)	0.033	0.495
Endoscopy	59.2	16 (-73)	23.5 (-60.3)	40 (-32.4)	<0.001	<0.001
Interventional radiology	12.1	5 (-58.7)	7.5 (-38.0)	9.2 (-24.3)	<0.001	0.026

The airway procedural data are reported in Table 5. In the PP phase, an ETT was used in 56.1% of cases and an LMA in 4.0%. In the EP phase, there was a significant increase in the percentage of cases utilizing ETT to 63.2% (P < 0.001). This increase was accompanied by a corresponding decrease in LMA usage which dropped to 0.8% in the EP phase (P < 0.001). A significant increase in documented RSII from 10.3% to 52.3% was also noted between the PP and EP phases (P < 0.001). In the R2 phase, RSII use had decreased but remained significantly elevated from the PP phase (23.1%, P < 0.001). The electronic medical record used in the anesthetic documentation at our institution provides an opportunity to document both that an RSII was performed and similarly that “mask ventilation was not attempted”. Due to variations in clinician documentation, both variables were evaluated to assess for changes in baseline practice. The number of cases in which it was documented that mask ventilation was not attempted increased from 16.4% to 76.5% between the PP and EP phases (P < 0.001). Like documented RSII use, the percentage of patients in which mask ventilation was not attempted decreased from the EP to R2 phases but remained increased at 40.0% of cases (P < 0.001). Also reflecting airway management recommendations, the percentage of intubations using VL increased from 24.6% to 79.6% between the PP and EP phases (P < 0.001). In the R2 phase, VL was used in 35.2% of intubations which continued to be a significant increase from PP use (P < 0.001). The percentage of fiberoptic intubations did not change throughout the pandemic period.

**Table 5:** Distribution of airway management procedures and individuals performing procedures. Values are n (percentage of total cases, total intubations, or total intubations by site). CRNA: Certified Registered Nurse Anesthetist; EP: Early Pandemic Phase; MAC: Monitored Anesthesia Care; OR: Operating Room; PP: Pre-Pandemic Phase; PUI: Patient Under Investigation; R1: Recovery 1 Phase; R2: Recovery 2 Phase; SRNA: Student Resident Nurse Anesthetist; STC: Shock Trauma Center; UMMC: University of Maryland Medical Center.

	Phase				P value	
	PP	EP	R1	R2	PP vs EP	PP vs R2
Airway procedure details						
Endotracheal tube	3142 (56.1)	993 (63.2)	952 (65.6)	3688 (58.8)	<0.001	0.003
Laryngeal mask airway	228 (4)	12 (0.8)	7 (0.5)	216 (3.4)	<0.001	0.072
Video laryngoscopy	827 (24.6)	800 (79.6)	625 (65.2)	1374 (35.2)	<0.001	<0.001
Rapid sequence intubation	347 (10.3)	526 (52.3)	490 (51.1)	903 (23.1)	<0.001	<0.001
Mask ventilation not attempted	553 (16.4)	769 (76.5)	714 (74.5)	1561 (40)	<0.001	<0.001
Fiberoptic	41 (1.2)	10 (1.0)	8 (0.8)	37 (0.9)	0.564	0.267
MAC	654 (11.7)	141 (8.9)	166 (11.4)	736 (11.7)	0.003	0.923
Airway in place at case start	485 (8.7)	270 (17.2)	194 (13.4)	825 (13.2)	<0.001	<0.001
Individual performing intubation						
Attending	334 (9.9)	71 (7.1)	66 (6.9)	377 (9.7)	0.006	0.715
CRNA	1013 (30.1)	539 (53.6)	428 (44.6)	1388 (35.6)	<0.001	<0.001
Trainee (fellow/resident/SRNA)						
UMMC-main ORs	1232 (60.7)	279 (45.4)	290 (47.5)	1272 (52.0)	<0.001	<0.001
UMMC-all locations	1510 (56.7)	306 (41.6)	319 (43.5)	1533 (50.0)	<0.001	<0.001
STC	385 (54.5)	58 (21.6)	111 (49.1)	468 (55.9)	<0.001	0.587
Extubated in OR	2597 (83.3)	799 (81.5)	785 (83.8)	3058 (83.7)	0.202	0.641

The provider recorded as performing the airway procedure was also investigated and reported in Table 5. Compared to PP values, in the EP phase, there was a significant decrease in the percentage of airway procedures performed by attending anesthesiologists and trainees. By the R2 phase, the percentage of airway procedures performed by attending anesthesiologists had rebounded to 9.7%. Airway procedures performed by trainees at UMMC decreased from 60.7% to 45.4% between PP and EP phases (P < 0.001). In the R2 phase, trainees at UMMC performed 52.0% of airway procedures representing a smaller but still significant decrease from PP values (P < 0.001). At STC, airway procedures performed by trainees decreased from 54.5% to 21.6% between PP and EP phases (P < 0.001). By the R2 phase, airway procedures performed by trainees at STC had rebounded to 55.9%, which was not significantly different from the PP phase. There was no statistically significant change in the number of patients undergoing extubation in the OR after undergoing intubation as a part of their anesthetic plan across the phases.

Finally, a review of ACGME index cases for the 2020 and 2021 academic years encompassing the EP, R1, and R2 phases demonstrated that all trainees met or exceeded the ACGME minimum targets.

## Discussion

The initial impact of changes in case and procedural volumes on our program was primarily limited to the EP and R1 phases lasting a combined 10 weeks. The drop in case volume at UMMC and STC closely mirrored that reported in the US [28] and elsewhere [6,7,15]. The impact of the COVID-19 pandemic on our anesthesiology residents varied between trainees depending on what rotations they were scheduled to complete during the most heavily impacted pandemic phases. For example, residents assigned to complete their first pediatric anesthesia rotation during the EP phase saw a significant reduction in case exposure compared to the previous year. Of the most affected case types, only pediatric, cardiac, and thoracic cases are represented on the ACGME requirement list. While the overall case volume is sufficient to allow for adjustments in scheduling to compensate for missed exposures, there is likely significant variability between training institutions, where some may be challenged in making the ACGME targeted minimums.

The small increase observed in the percentage of cases being performed by residents coincided with a change in the coverage model. First, all residents that were assigned to off-site rotations, pain clinic, and pre-surgical clinic were reassigned to UMMC during a portion of the EP phase to maintain training while those sites were closed. All OR-based, intensive care unit (ICU), post-anesthesia care unit (PACU), and regional anesthesia and acute pain medicine rotations were continued. One resident and one attending were dedicated to covering the airway service, and one extra resident and faculty were assigned to labor and delivery due to an unchanged clinical volume and increased workload from pandemic-related changes such as expanded PPE usage. Secondly, as elective cases decreased, changes were made to prioritize residents over non-trainee staff to maintain the clinical experience. Unfortunately, this increase in the overall percentage of cases performed was not significant until the R1 phase.

Early in the pandemic, consensus guidelines regarding airway management in patients with COVID-19, as well as those patients requiring interventions without prior testing, gave recommendations for intubation, mask ventilation, supraglottic airway usage, and induction of anesthesia to minimize provider exposure [21,23,24]. Since these are considered significant AGPs, the guidelines were universally recommended during the transition to the current endemic phase for all patients requiring intubation in the OR or requiring airway management in other settings regardless of prior SARS-CoV-2 testing results [22]. This included the use of RSII for all patients, prioritization towards the more experienced laryngoscopist, avoidance of supraglottic airways, and emphasis on VL when required for advanced airway management [25]. Our program largely adopted these recommendations early in the pandemic. There was a significant change in airway management practice during the EP phase, with a shift towards VL with less LMA usage and an increased use of RSII. This was accompanied by a corresponding decrease in attempts at mask ventilation before intubation.

For ongoing training in airway management, as noted by Cook et al., there will be challenges to establishing early competence in basic airway skills, particularly for DL, manual ventilation, insertion/confirmation of supraglottic airways, and maintenance of the airway with oral/nasopharyngeal airways if we continue to rigidly follow these guidelines [22]. In our program, which includes not only anesthesiology trainees, but others from critical care and emergency medicine, practice has expanded to allow for more trainee involvement. There has also been a concomitant increase in usage of DL and manual ventilation during induction of anesthesia. Although the practice has not completely returned to that before the pandemic, there is no longer a strict adherence to the original recommendations that were largely based on consensus opinion. This may be due to more recent work suggesting a lower risk of exposure during tracheal intubation and extubation than previously assumed [29] and increased comfort with the level of protection offered by continued PPE usage and pre-screening for the presence of SARS-CoV-2.

One of the interesting findings regarding airway management in our study relates to the recommendation that the most experienced provider manage the airway to reduce the time to intubation and improve the first pass success rate. We found that the percentage of intubations by the attending anesthesiologist was significantly reduced from 9.9% to 7.1% comparing the PP to EP phases and returned to baseline by the R2 phase. While there was a moderate drop in the percentage of intubations for residents at UMMC, the STC trainees showed a more significant drop. The total number of intubations done by trainees was further impacted due to the reduction in total case volume. Since many non-anesthesiology trainees were absent from the operating theater due to the COVID-19 responses to their own programs, this had a greater impact on their availability for training at STC, where they represent a greater percentage of total trainees. This recovered back to PP phase numbers at STC but did not fully return to baseline for UMMC. In total, trainees continued to be involved in airway management in the OR through all phases of the COVID-19 pandemic, although non-anesthesiology trainees were likely the most impacted by the lost exposure.

One additional recommendation early in the pandemic was an increased emphasis on regional anesthesia [19,22,30]. This recommendation was based on resource utilization, avoidance of aerosolizing procedures, and OR efficiency. We found that the use of regional during the PP phase had increased significantly from the prior year, making the drop during the EP and R1 phases even more profound. Compared to the same time period in 2019, there was a 42.3% and 54.5% drop in regional procedures during the EP and R1 phases, respectively. This decrease is even more significant when taking the overall drop in case volume into account. The cancellation of elective procedures during the EP phase may have directly impacted this metric since they make up a significant portion of the cases utilizing regional anesthesia. Clearly, the exposure to regional anesthesia for the trainee population was severely impacted during the early pandemic phases but has largely rebounded to near baseline during the current endemic phase, even with subsequent waves of COVID-19 cases entering the healthcare system.

Limitations

Our study is limited to the experience of a single institution. Local guidelines, policies, and COVID-19 prevalence are likely to have had a lesser or greater impact on individual programs around the world. In addition, we only looked at the first major wave of cases affecting our institution, although subsequent waves have identified a significant increase in COVID-19 cases. These subsequent waves, however, did not result in alterations to elective and emergent surgical case volume and thus likely had less impact on anesthesiology resident training. Overall, the results may not be generalizable to other academic and private hospital systems having a different response to the pandemic. In addition, using an electronic medical record is subject to data entry errors and may have resulted in missed, underrepresented, or misclassified data.

## Conclusions

As the COVID-19 pandemic continues to evolve, it remains to be seen what lasting implications it will have on anesthesia training. As we have found, the initial response to a pandemic of this type will impact surgical case volumes, procedural counts, and other aspects of anesthesia training. As our procedures and resource availability improved, the ability to resume training operations has returned to normal with increased comfort in providing care in the setting of a pandemic, with all trainees meeting or exceeding ACGME minimum case targets upon completion of their training during the pandemic. At the same time, elements that were adopted during the pandemic, such as increased use of VL and RSII, have yet to return to prior levels and may represent the “new normal”.
